# Differential Inhibitory Effects of Curcumin Between HPV+ve and HPV–ve Oral Cancer Stem Cells

**DOI:** 10.3389/fonc.2018.00412

**Published:** 2018-09-26

**Authors:** Nasreen Bano, Manisha Yadav, Bhudev C. Das

**Affiliations:** ^1^Dr. B.R. Ambedkar Center for Biomedical Research, University of Delhi, New Delhi, India; ^2^Stem Cell & Cancer Research Lab, Amity Institute of Molecular Medicine & Stem Cell Research, Amity University, Noida, India

**Keywords:** cancer stem cells (CSCs), oral squamous cell carcinoma (OSCC), curcumin, human papillomavirus (HPV), SP cells (side population), stemness, orospheres, micro-RNA (miRNA)

## Abstract

**Aim:** To investigate the role of a herbal antioxidative compound curcumin on cell proliferation, orosphere formation and miRNA-21 expression in HPV16+ve/–ve oral cancer stem cells.

**Materials and Methods:** Oral cancer stem cells were isolated from HPV+ve/HPV–ve oral cancer cell lines by FACS and stemness markers. MTT, spheroid assay and qRT-PCR were employed to examine the effects of curcumin.

**Results:** Curcumin treatment in micromolar concentration (0–50 μM) demonstrated significant differential inhibition in CSC proliferation, orosphere formation and miRNA-21 expression in a dose dependent manner, the effect being highly pronounced in HPV positive CSCs.

**Conclusion:** The strong and dose-dependent inhibitory effects of curcumin on cell proliferation, stemness and miRNA appear to be due to its chemosensitizing and anticancer effects on OSCC-CSCs.

## Introduction

Oral squamous cell carcinoma (OSCC) is the most common subtype of head and neck squamous cell carcinoma (HNSCC) and ranks sixth in the cancer incidence worldwide. A significant proportion of oral cancers have been shown to contain specific high risk (HR) human papillomavirus (HPV) infection (1, 2) besides smoking and alcohol consumption.

Emerging literature suggests presence of a small sub-population of cancer stem cells (CSCs), a rare undifferentiated fraction of tumor cells that are intrinsically resistant to chemo-radiotherapy (3), reconstitute the tumor, and subsequently cause metastasis and tumor recurrence (4, 5). Altered expressions of microRNAs (miRNAs) are associated with clinical prognosis of tumor, metastasis, angiogenesis, drug resistance and are more importantly considered to be the regulators of CSC phenotype and their functions (6).

Despite emergence of various novel therapeutic strategies, OSCC patients have approximately only 50–55% 5-year survival rate (7). The extent of this problem mandates the need for novel therapeutic agents, specifically for chemoprevention.

Curcumin, a pharmacologically safe herbal compound is known for its anticancer effects, including inhibition of proliferation and angiogenesis, induction of apoptosis and increased chemo-radiosensitivity (8–11).Recent studies have demonstrated curcumin as a potent inhibitor of AP-1, NF-kB, and HPV in cervical and oral cancer (11–15) but its effects on oral cancer and CSCs with or without HPV infection are unknown. Therefore, the present study was performed to clarify the effects of curcumin in OSCC-CSCs.

## Materials and methods

### Cell lines

The present study has been carried out using three cell lines, one HPV16+ve OSCC cell line, UD-SCC-2 (gift from Dr. Henning Bier, University of Dusseldorf, Germany) and two HPV–ve OSCC cell lines, UPCI:SCC131 and UPCI:SCC84 (kind gift from Dr. Susanne M. Gollin, University of Pittsburgh, Pittsburgh, USA). The UD-SCC2 cell line was grown in RPMI 1640 medium while UPCI: SCC131 and UPCI: SCC84 cells were grown in DMEM medium (Sigma Aldrich) supplemented with 10% Foetal bovine serum, 1% Penicillin and Streptomycin (penstrap) (Gibco, Thermo Fisher Scientific Inc., MA USA) at 37°C in a humidified atmosphere containing 5% CO_2_ at BSL2 level.

Curcumin was obtained from Sigma Chemicals (St. Louis, MO, cat no. C1386) and was dissolved in DMSO to make a 50 mM stock solution and was further diluted in the medium immediately before use.

### Analysis of SP and non-SP cancer cells by FACS

SP analysis was based on the previously described method using Hoechst 33342 dye and its inhibitor verapamil as performed in our laboratory with slight modifications (16, 17). Analysis and sorting was performed on FACS Aria III (BD Biosciences) using Diva Software.

### Protein extraction and western blotting

Cell extracts from SP, NSP, Parental cells (unsorted original cell lines) from OSCC cell lines were prepared by the method of Dignam (18) with minor modification as described earlier (1). The primary antibodies used were: β-actin (C-11), Oct-3/4 (sc-5279), and SOX2 (sc-17320), (Santa Cruz Biotechnology, USA).

### Cell proliferation by MTT assay

Parental and NSP cells (5 × 10^3^ cells/well) were seeded in quadruplicates in 96 well plates. After 24 h, cells were incubated in the absence and presence of varying concentrations of curcumin (0–50 μM) for 24 h at 37°C. To check the effect of curcumin on the viability of spheroids, SP cells were seeded in 24-well ultra-low attachment plates and grown as spheroids. After 3 days, curcumin (0–50 μM) was added to the wells and MTT assay was performed as described previously (15).

### Sphere formation assay

Sphere formation assay from sorted SP cells was performed using the procedure described earlier (17). Three days after seeding, the orospheres at their stable growth condition were treated with 0–50 μM of curcumin for 24 h and after 7–10 days, the numbers of orospheres were photographed and counted (17).

### RNA extraction and quantitative real-time PCR

The total RNA was extracted from SP cells derived from all three cell lines using mirVana™ miRNA isolation kit (Ambion, USA) according to manufacturer's protocol after 24 h of 30 μM curcumin treatment. Real-Time PCR was performed using primers hsa-miR-21 and RNU6B as a reference control and 0.1% DMSO as vehicle control using the TaqMan universal mastermix kit (Applied Biosystem, USA) as described previously (19).

### Statistical analysis

The data analysis was performed using Graph Pad Prism (version 6.0) and image J software. Two-way ANOVA followed by Bonferroni *post-hoc* was applied. *P* < 0.05 is considered as statistically significant.

## Results

### Side population contains CSCs in HPV+ve and HPV–ve OSCC cell lines

**Figure 1:**
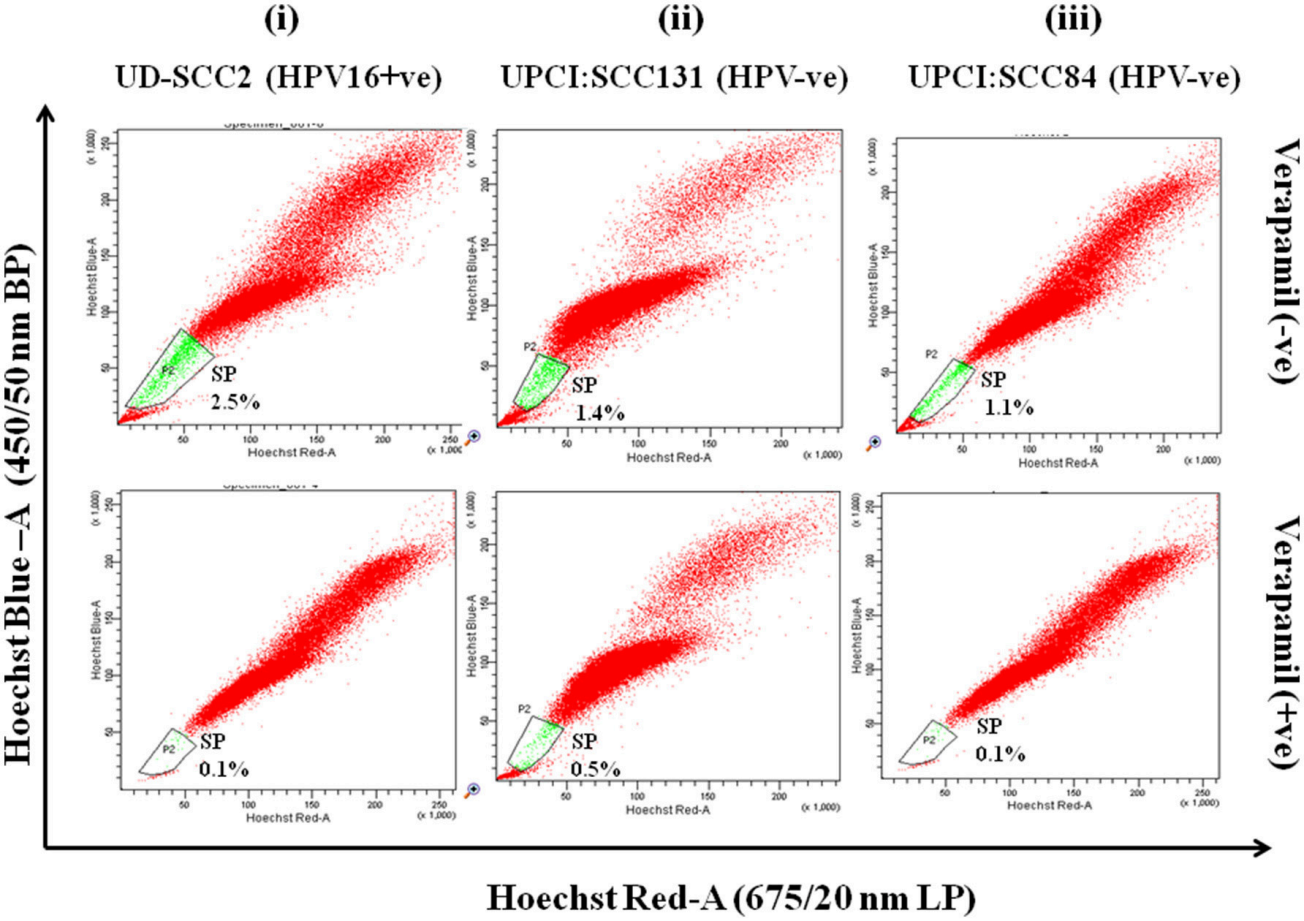
**(i–iii)** Flow cytometric (FACS) analysis of SP cells in OSCC cell lines A. Flow cytometric analysis of side population (SP) in **(i)** UD-SCC2 (HPV16+ve), **(ii)** UPCI: SCC131 (HPV–ve) and **(iii)** UPCI:SCC84 (HPV–ve) OSCC cell lines. OSCC cells were stained with Hoechst 33342 dye alone or in the presence of verapamil and analyzed by flow cytometry measuring Hoechst blue vs. Hoechst red fluorescence. The SP was gated and represented as a percentage of the whole viable cell population following propidium iodide exclusion.

Flow cytometric analysis was performed in all three OSCC cell lines for isolation of side population as CSCs. SP cells occupied 2.5, 1.4, and 1.1% of the total cells in UD-SCC2, UPCI:SCC131 and UPCI:SCC84 (Figure 1-upper panel) cell lines and when pre-incubated with its inhibitor verapamil, the percentage of SP cells shrank to 0.1, 0.5, and 0.1% of total cells in UD-SCC2, UPCI:SCC131, and UPCI:SCC84, respectively (Figure 1-lower panel). The cells outside the gated area represent the non-side population (NSP).

### Expression of cancer stemness markers in HPV+ve/HPV–ve oral CSCs

We observed that upregulated expression of stemness markers Oct-4 and Sox-2 in SP cells was significantly higher when compared with that of Parental and NSP cells in both HPV+ve/HPV–ve cells and this relative increased expression level is more prominent in HPV16+ve cells as compared to that of HPV–ve cells (see Supplementary Figures 1i,ii).

### Differential orosphere formation ability by HPV+ve/HPV–ve oral CSCs

Sorted SP cells from three OSCC cell lines grew as three-dimensional spheres called orospheres. However, UD-SCC2-SP cells (HPV16+ve) formed a high degree of loose and less rounded clusters of orospheres than those observed as compact and rounded orospheres in UPCI:SCC131-SP (HPV–ve) and UPCI:SCC84-SP (HPV–ve) cells with SFE (sphere forming efficiency) (UD-SCC2-SFE, 0.325%; UPCI:SCC131-SFE-, 0.235%; UPCI:SCC84, 0.21%; see Supplementary Figures 2A,B).

### Curcumin inhibits oral cancer stem cell growth

**Figure 2:**
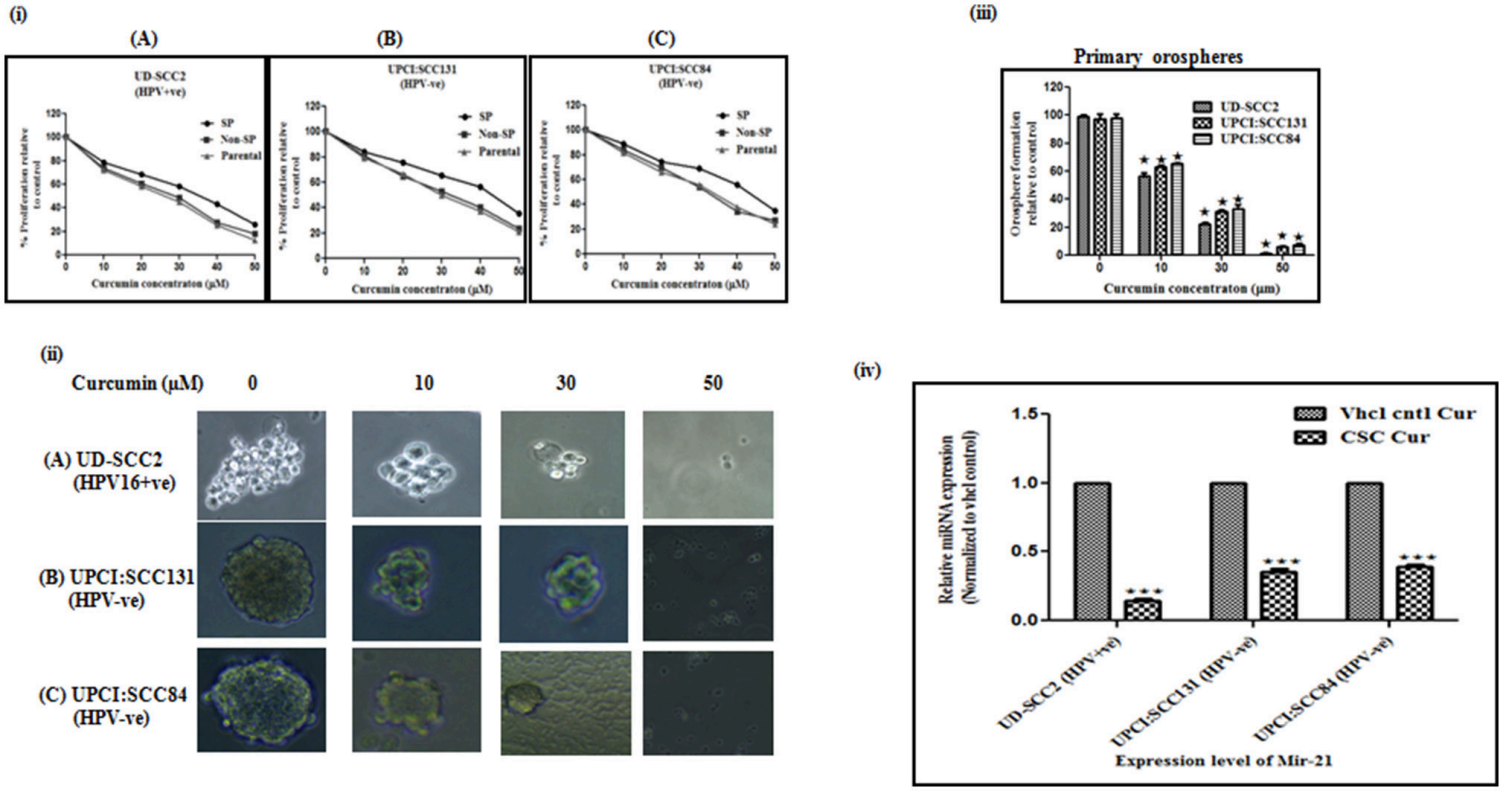
**(i–iv)** Curcumin inhibits cell proliferation rate, spheroid formation and miRNA-21 expression in oral cancer stem cells. **(i)** Cell proliferation rate: Parental, SP and NSP cells of (A) UD-SCC2 (HPV16+ve), (B) UPCI:SCC131 (HPV–ve), and (C) UPCI:SCC84 (HPV–ve) were incubated with increasing concentrations of curcumin (0–50 μM) for up to 24 h. and analyzed for cell proliferation rate. Curcumin treatment resulted in a significant dose dependent decrease in cell proliferation in all three cells when compared with untreated controls. Results are representative of three independent experiments. **(ii)** Spheroid formation ability: (A) CSCs from UD-SCC2 (HPV16+ve), (B) UPCI:SCC131(HPV–ve) and (C) UPCI:SCC84 cells were grown in low adherent plates and treated with increasing concentrations of curcumin (0, 10, 20, 30 and 50 μM) and performed spheroid assay to assess the effect of curcumin on orosphere forming ability. **(iii)** Primary sphere formation: Effect of curcumin on OSCC-SP spheroid formation ability was analyzed by counting spheroid and sphere forming efficiency was calculated and bar diagram was drawn. Curcumin treatment significantly inhibited orosphere formation in HPV+ve and HPV–ve OSCC-SP orospheres. The experiments were performed at least three times and data are presented here as mean ± standard errors. *P*-value is determined with respect to their vehicle controls (*p < 0.05). **(iv)** Downregulation of miRNA-21 expression: The expression level of miR-21 in orosphere forming CSCs of UD-SCC2 (HPV+ve) and UPCI:SCC131 and UPCI: SCC 84(HPV–ve) cell lines after 24 h of 30 μM curcumin treatment. RT-qPCR was used to detect miRNA expression and RNU6B was used to normalize data. Each set is normalized with their respective vehicle control. The experiments were performed at least three times and data are presented here as mean ± standard errors. ***P < 0.0001, *P < 0.01.

Curcumin significantly suppressed the proliferation of CSCs derived from both HPV+ve and HPV–ve cell lines in dose dependent manner (Figure 2i). Viability of SP cells derived from the OSCC cell lines was found to be higher than that of the NSP and parental cells. The effect of curcumin between HPV+ve and HPV–ve cells, indicated relatively a stronger cytotoxic effect on UD-SCC2 HPV+ve SP cells (IC_50_-36.21 μM) when compared to UPCI:SCC84 HPV–ve (IC_50_-45.12 μM)/UPCI:SCC131 SP cells (IC_50_-46.56 μM) as shown in Figures 2iA–C.

### Curcumin inhibits orosphere formation ability

Curcumin treatment significantly inhibited orosphere formation in a dose dependent manner in both HPV+ve/HPV–ve CSCs. However, this effect seems to be more pronounced in HPV+ve CSCs at 30 and 50 μM concentrations (Figure 2ii). Curcumin treatment progressively reduced primary orospheres in all three cell lines and it was observed that sphere formation at 50 μM concentration was very low in HPV–ve SP cells while it was almost nil in HPV+ve OSCC-SP cells (Figure 2iii).

### Curcumin significantly downregulated miRNA-21 expression in oral CSCs

Since at 50 μM concentration, sphere formation was very low in HPV–ve and almost nil in HPV+e SP cells, 30 μM was considered as optimal concentration for analysing miRNA-21 expression. The fold change expression level of miR-21 in CSCs of HPV+ve UD-SCC2, HPV–ve UPCI:SCC131, and UPCI:SCC84 cells were 0.14 (*p* = 0.001), 0.35 (*p* = 0.001), and 0.39 (*p* = 0.001) which were significantly different from those of vehicle counterparts (Figure 2iv).

## Discussion

A large number of studies indicate that CSCs are not only tumor initiators but also associated with aggressive metastasis, drug resistance, tumor recurrence. CSCs can reflux out the cytotoxic drugs due to the expression of multidrug-resistance linked ATP binding cassette (ABC) drug transporter protein, ABCG2 (3). Alternative treatment approaches like using natural product remedies as they are easily tolerated with no apparent/significant toxicity as compared to strong adverse effects of standard chemotherapeutic drugs. Curcumin is well-studied and reported to possess antiviral, anticancer and anti-inflammatory effects (11, 12, 20). It was observed that in preliminary trials, breast cancer patients receiving curcumin orally (500 mg twice a day) alongwith standard treatment showed better prognosis with less side effects (Das et al unpublished). microRNAs also play important role in the post-transcriptional regulation of genes and in regulating stem cell phenotype (21). Therefore, targeting CSCs along with relevant miRNAs with curcumin may provide a novel and more effective therapeutic approach for the eradication of CSCs.

We report here that oral CSCs isolated from both HPV+ve/HPV–ve OSCC cell lines show self-renewal property and orosphere formation ability. It is further shown that curcumin causes loss of proliferation of oral cancer and CSCs. Our findings are consistent with previous studies on breast (22), colon (9), cervical (11), and ovarian cancer (23). Curcumin treatment inhibited cell viability more effectively in HPV+ve and HPV–ve parental and NSP than that of CSCs. Less cytotoxic effect on CSCs could be attributed to limited penetration or inadequate availability of curcumin to SP cells. Similar results were also observed in earlier studies (23, 24). However, inhibition of proliferation was found to be highly pronounced in HPV+ve CSCs than that of HPV–ve CSCs. Higher antiproliferative effects in HPV16+ve CSCs observed in the present study gains support from earlier reports that showed strong anti-HPV effect of curcumin in cervical and oral cancer (12, 15, 25). The suppression of HPV 16 E6/E7 oncoproteins by an antisense method and curcumin in HPV16+ve cervical and oral cells have been shown to restore apoptosis as well as re-appearance of p53 (25–27). Studies reporting the effect on resistance-linked ATP-binding cassette transporters revealed specific inhibition of ABCG2 by curcumin (11, 28, 29). In a recent study, curcumin has been shown to inhibit ABCG2 by binding to a site different from the standard inhibitor, FTC binding site (11). Interestingly, HPV+ve UD-SCC2 cells formed relatively loose and less rounded orospheres than those of HPV–ve UPCI:SCC84 and UPCI:SCC131 cells, and this probably could be the reason for better penetration and more effectiveness of curcumin in HPV+ve orospheres than HPV–ve's.

We further analyzed the effect of curcumin on sphere formation which is indicative of tumorigenic potential of CSCs. It was observed that curcumin (50 μM) significantly abrogated sphere forming ability of HPV–ve CSCs while it was almost nil in HPV+ve CSCs. Our data corroborates with earlier studies in cervical, breast, esophageal and colon cancer (11, 15, 28). Curcumin treatment inhibits sphere formation by inhibiting the ABCG2 transporter and cyclin D1 in SP cells leading to G_0_-G_1_ arrest (30, 31).

A strong anti-miR-21 activity of curcuimin has been shown in pancreatic, colon and esophageal cancer leading to inhibition of invasion and metastasis (32–34). Our results demonstrated downregulation of miR-21 expression upon curcumin treatment in both HPV+ve/HPV–ve CSCs but with higher effects in HPV+ve CSCs.

Our results show an evidence that curcumin can sensitize the CSCs by inhibiting cell growth *in vitro*, abrogating sphere formation and downregulating miR-21 expression more effectively in HPV+ve oral CSCs thus making curcumin as a strong candidate for therapeutic applications. Therefore, use of curcumin may make the cancer treatment more effective when used along with standard anticancer drugs or radiation, if not curcumin alone. Further investigations are needed to determine the solubility and bioavailability (35), mechanism(s) of action and targets of curcumin for better understanding of its role in deciphering its therapeutic effects.

## Ethics statement

The study was ethically approved by the Institutional Ethics committee of Dr. B. R. Ambedkar Center for Biomedical Research, University of Delhi, India (ACBR/09/13/IHEC/73).

## Author contributions

BD has conceptualized, designed, and supervised the study, interpreted the results and corrected the manuscript and communicated. NB has conducted all experiments collated and analyzed the data and prepared table and figures and written the manuscript. MY has helped in conducting experiments and writing and revising the manuscript. BD and MY have given administrative, technical and material support.

### Conflict of interest statement

The authors declare that the research was conducted in the absence of any commercial or financial relationships that could be construed as a potential conflict of interest.

## Supplementary material

Supplementary Figure 1 OSCC express CSC markers: **(i)** Western blot analysis of cancer stem cell markers from protein extract of sorted SP, NSP and parental cells from UD-SCC2 HPV16+ve, UPCI:SCC131 (HPV–ve)and UPCI:SCC84 (HPV–ve) cells. A total of 25 μg protein extracts each from SP, NSP, and parental cells were separated on a 10% SDS-PAGE, electrotransferred on PVDF membrane and probed. To confirm equal protein loading, the membranes were reprobed for β-actin expression. **(ii)** The relative normalized fold change in the protein is expressed as the mean ± SD of three independent experiments. ^***^*P* < 0.001, ^**^*P* < 0.01, ^*^*P* < 0.05 for UD-SCC2 HPV16+ve, UPCI:SCC131 (HPV–ve)and UPCI:SCC84(HPV–ve) cells.

Supplementary Figure 2 Functional characterization of SP cells present in OSCC cell lines. (**A**i,**A**ii) Assessment of orosphere forming ability of SP cells. Representative photomicrograph of orosphere formation with sorted SP in low adherence defined Serum free media (DSFM) in (i) UD-SCC2 HPV16+ve, (ii) UPCI:SCC131 (HPV–ve) and (iii) UPCI:SCC84 (HPV–ve) cells (magnification 40X) and **(B)**. Spheres with 0.75 mm diameter were counted after 10 days. The percentage of sphere forming cells was calculated by dividing the number of orospheres formed with the number of cells seeded. The experiments were performed at least three times and data are presented here as mean ± standard errors. UD-SCC2-SFE, 0.325%; UPCI:SCC131-SFE-, 0.235%; UPCI:SCC84, 0.21%.
