# Supplementary figures and images for: Differential Inhibitory Effects of Curcumin Between HPV+ve and HPV–ve Oral Cancer Stem Cells
Source: Front Oncol. 2018 Sep 26;8:412. doi: 10.3389/fonc.2018.00412 (PMC6168628; doi:10.3389/fonc.2018.00412)

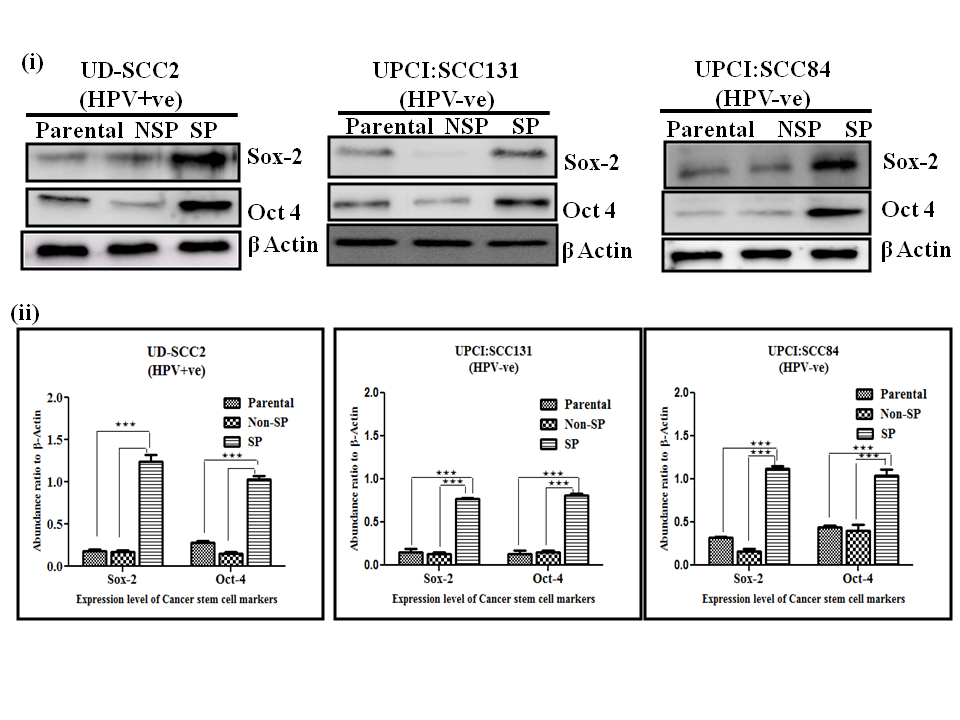

Supplement: Supplementary Figure 1 — OSCC express CSC markers: (i) Western blot analysis of cancer stem cell markers from protein extract of sorted SP, NSP and parental cells from UD-SCC2 HPV16+ve, UPCI:SCC131 (HPV–ve)and UPCI:SCC84 (HPV–ve) cells. A total of 25 μg protein extracts each from SP, NSP, and parental cells were separated on a 10% SDS-PAGE, electrotransferred on PVDF membrane and probed. To confirm equal protein loading, the membranes were reprobed for β-actin expression. (ii) The relative normalized fold change in the protein is expressed as the mean ± SD of three independent experiments. ***P < 0.001, **P < 0.01, *P < 0.05 for UD-SCC2 HPV16+ve, UPCI:SCC131 (HPV–ve)and UPCI:SCC84(HPV–ve) cells. [file Image_1.TIF]

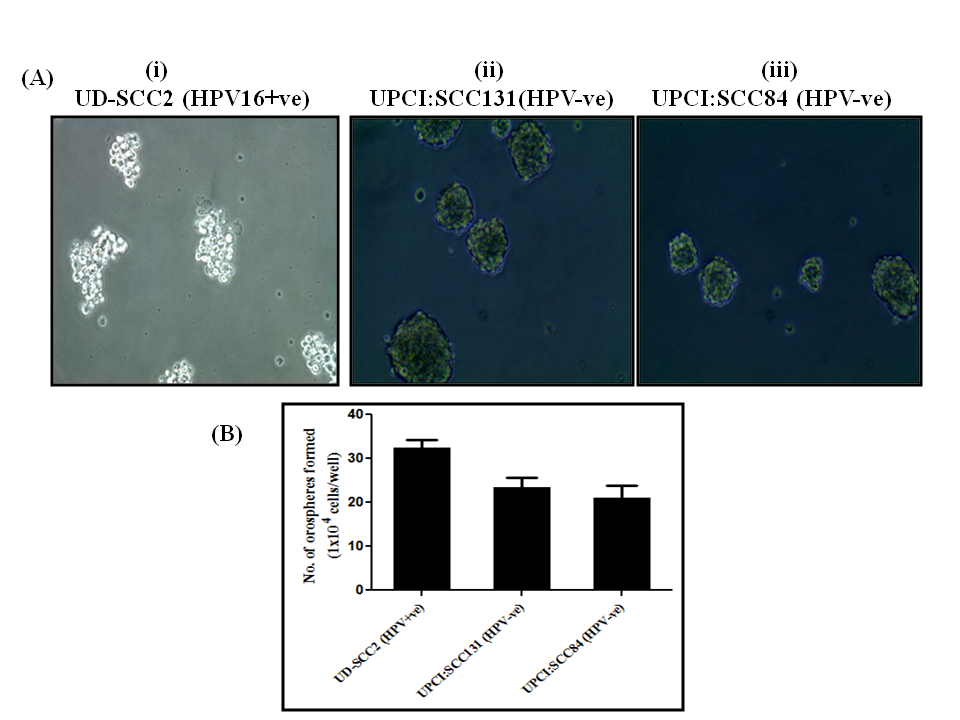

Supplement: Supplementary Figure 2 — Functional characterization of SP cells present in OSCC cell lines. (Ai,Aii) Assessment of orosphere forming ability of SP cells. Representative photomicrograph of orosphere formation with sorted SP in low adherence defined Serum free media (DSFM) in (i) UD-SCC2 HPV16+ve, (ii) UPCI:SCC131 (HPV–ve) and (iii) UPCI:SCC84 (HPV–ve) cells (magnification 40X) and (B). Spheres with 0.75 mm diameter were counted after 10 days. The percentage of sphere forming cells was calculated by dividing the number of orospheres formed with the number of cells seeded. The experiments were performed at least three times and data are presented here as mean ± standard errors. UD-SCC2-SFE, 0.325%; UPCI:SCC131-SFE-, 0.235%; UPCI:SCC84, 0.21%. [file Image_2.TIF]
